# Mobile Diabetes Intervention Study of Patient Engagement and Impact on Blood Glucose: Mixed Methods Analysis

**DOI:** 10.2196/mhealth.9265

**Published:** 2018-02-02

**Authors:** Charlene Connolly Quinn, Erin C Butler, Krystal K Swasey, Michelle D Shardell, Michael D Terrin, Erik A Barr, Ann L Gruber-Baldini

**Affiliations:** ^1^ Department of Epidemiology and Public Health University of Maryland School of Medicine Baltimore, MD United States; ^2^ Department of Emergency Medicine Wellspan York Hospital York, PA United States; ^3^ National Institute on Aging Baltimore, MD United States

**Keywords:** mobile health, diabetes, engagement, randomized clinical trial, qualitative, digital health

## Abstract

**Background:**

Successful treatment of diabetes includes patient self-management behaviors to prevent or delay complications and comorbid diseases. On the basis of findings from large clinical trials and professional guidelines, diabetes education programs and health providers prescribe daily regimens of glucose monitoring, healthy eating, stress management, medication adherence, and physical activity. Consistent, long-term commitment to regimens is challenging. Mobile health is increasingly being used to assist patients with lifestyle changes and self-management behaviors between provider visits. The effectiveness of mobile health to improve diabetes outcomes depends on patient engagement with a technology, content, or interactions with providers.

**Objectives:**

In the current analysis, we aimed to identify patient engagement themes in diabetes messaging with diabetes providers and determine if differences in engagement in the Mobile Diabetes Intervention Study (MDIS) influenced changes in glycated hemoglobin A_1c_ (HbA_1c_) over a 1-year treatment period (1.9% absolute decrease in the parent study).

**Methods:**

In the primary MDIS study, 163 patients were enrolled into 1 of 3 mobile intervention groups or a usual care control group based on their physician cluster randomization assignment. The control group received care from their physicians as usual. Participants in each intervention group had access to a patient portal where they could record monitoring values for blood glucose, blood pressure, medication changes, or other self-management information while also assigned to varying levels of physician access to patient data. Intervention participants could choose to send and receive messages to assigned certified diabetes educators with questions or updates through the secure Web portal. For this secondary analysis, patient engagement was measured using qualitative methods to identify self-care themes in 4109 patient messages. Mixed methods were used to determine the impact of patient engagement on change in HbA_1c_ over 1 year.

**Results:**

Self-care behavior themes that received the highest engagement for participants were glucose monitoring (75/107, 70.1%), medication management (71/107, 66.4%), and reducing risks (71/107, 66.4%). The average number of messages sent per patient were highest for glucose monitoring (9.2, SD 14.0) and healthy eating (6.9, SD 13.2). Compared to sending no messages, sending any messages about glucose monitoring (*P=*.03) or medication (*P*=.01) led to a decrease in HbA_1c_ of 0.62 and 0.72 percentage points, respectively. Sending any messages about healthy eating, glucose monitoring, or medication combined led to a decrease in HbA_1c_ of 0.54 percentage points compared to not sending messages in these themes (*P*=.045).

**Conclusions:**

The findings from this study help validate the efficacy of the mobile diabetes intervention. The next step is to determine differences between patients who engage in mobile interventions and those who do not engage and identify methods to enhance patient engagement.

**Trial Registration:**

ClinicalTrials.gov: NCT01107015; https://clinicaltrials.gov/ct2/show/NCT01107015 (Archived by WebCite at http://www.webcitation.org/6wh4ekP4R)

## Introduction

Type 2 diabetes is a growing national health concern affecting an estimated 10% of the US population [1]. It is a costly disease that requires an intricate self-management regimen including regular self-glucose monitoring, healthy eating, exercise, regular physician examinations, and specialist visits. Many patients, however, miss recommended screenings and lack diabetes self-management education (DSME) leading to higher rates of poor glycemic management and associated complications [2-7].

Several studies have previously documented the efficacy of lifestyle modifications and drug therapy to prevent and treat type 2 diabetes, with many finding that lifestyle modifications are more effective at long-term prevention compared to metformin therapy [5,8-10]. The Action to Control Cardiovascular Risk in Diabetes (ACCORD) study suggested that intensive drug therapy could increase risk of adverse events and death, although mechanisms of the adverse events remain unknown [11-13].

One approach to facilitate self-management and lifestyle changes is behavior intervention technology, which uses technology and mobile health to target specific short- and long-term treatment and management goals [14]. There have been numerous phone-, text-, and Web-based intervention studies in recent years that demonstrate mixed impact when compared to traditional phone call or face-to-face intervention strategies. One personal digital assistant–based intervention found some improvement in glycated hemoglobin A_1c_ (HbA_1c_) management during a 9-month study, and another found improvement with an intervention based on regularly scheduled telephone calls depending on patient risk level [15,16]. Although some short message service interventions have been shown to help prevent and manage diabetes [17,18], other studies have found that strictly phone-based applications have minimal impact on glycemic management compared to traditional intervention methods [19]. Furthermore, many studies on Web-based interventions, including components for tracking blood glucose readings, medications, diet, exercise, and weight loss through an online portal system, had varying degrees of success at helping participants lose weight and improve glycemic management [20-24].

A promising future direction of mobile diabetes management may be an integrated system that uses multiple means of access via Web portals or mobile apps and provides people with feedback based on their tracking data [7,21,25-27]. A particularly effective component of many recent studies is patient interaction with certified diabetes educators (CDEs) via phone, email, or other messaging systems. Regardless of medium, patient engagement and the ability to communicate with a diabetes educator helped improve outcomes across a variety of mobile health interventions [23,28-32]. Yet, less than 20% of currently available diabetes management applications have a motivational feedback component to them [32]. These cost-effective, easy-to-implement measures could help patients avoid expensive hospitalizations and diabetes complications by allowing them to manage their diabetes at home. Feedback components may also increase the efficiency for primary care physicians who manage patients with diabetes most often [29].

The messaging component of studies such as the DiabetesCoach intervention [31] show that patients are responsive to both automated and personalized messages, and an individualized, personal message option is effective at helping reach a given treatment outcome. However, further research into the impact of patient engagement and best practices to engage patients is needed before standards of care can be amended [32]. In this study, we identified patient engagement messages and assessed patient engagement in the Mobile Diabetes Intervention Study (MDIS) to determine if differences in engagement were related to changes in HbA_1c_.

## Methods

### Study Design and Eligibility

A detailed description of the Mobile Diabetes Intervention Study was published previously [33]. The study was a cluster-randomized clinical trial including 26 primary care physician groups across 4 geographic areas of Maryland. Randomization took place at the practice level to avoid contamination among physicians regarding care of their patients.

Eligible patients followed physician randomization assignment. Inclusion criteria for patients included diagnosis of type 2 diabetes at least 6 months prior to enrollment in the study, HbA_1c_ ≥7.5%, and age 18 to 64 years. Patients who were uninsured or Medicare or Medicaid beneficiaries were not included. Baseline data was collected from all participants, including demographic information, health history, current health status (including HbA_1c_) and medications, risk factors for complications associated with poor diabetes management, and lifestyle and self-management behaviors.

The MDIS enrolled 163 patients across 3 intervention groups and 1 control group. The control group received care from their physicians as usual. Participants in each intervention group had access to a patient portal where they could record self-care behavior while also assigned to varying levels of physician access to patient data. In the most complex intervention group, physicians could review raw patient data, see analyzed patient data reports every 3 months, and make treatment recommendations based on these summaries. All patients received their choice of 1 of 2 smartphones with an unlimited 1-year data plan as well as a OneTouch Ultra 2 (LifeScan Inc) glucose meter and enough testing supplies for the duration of the 1-year study.

For this secondary analysis of the MDIS, group 1 data was not evaluated because control patients were not able to message their providers.

### Patient Engagement

In addition to tracking information related to self-management of their diabetes, the secure patient portal allowed participants to communicate with CDEs throughout the study. When patients input data into the system, the computer would automatically generate feedback messages with encouragement or advice based on recently recorded data. For example, if a participant input a low blood glucose value, the system would provide a feedback message such as “This blood sugar is low! Eat 15 grams of carbs and recheck in 15 minutes.” Additionally, the data would be reviewed by the patient’s assigned CDE who could provide feedback intermittently. Most patients used the portal messaging system to communicate with educators over the course of the study, seeking advice, feedback, and answers to questions; however, using the messaging feature was not required for patients, and there was no set schedule of communication as part of the study intervention. The portal contained a variety of diabetes education materials including information on healthy eating, counting carbohydrates, being active, self-monitoring blood glucose, medications, and coping with and adjusting to living with diabetes.

Patients covered a wide variety of content in their messages to the CDEs from asking questions about healthy eating to changing medications to optimizing their medication schedule. To investigate the association of patient engagement with improved patient outcomes observed in previous studies [23,28-32], we evaluated patient engagement in our study through a qualitative analysis of messages sent through the secure patient portal.

For this analysis, we used the grounded theory approach [34] to analyze patient messages. As its name suggests, the theory is grounded in the observation of qualitative data and is used (for the purpose of this study) to categorize the data into core concepts. Based on review of a few sample patients, we created a coding scheme based on the 7 self-care behaviors for healthy living recommended by the American Association of Diabetes Educators (AADE) and the American Diabetes Association (ADA) [35,36]. After a pilot coding of 2 complete patient files, additional codes to account for patient-reported motivation and learning as well as general discussion about diet, medication, or self-monitoring of blood glucose were added (27 codes). Patient messages were then coded by EB and CQ (team members) based on the 27 codes developed for this project, with the appropriate codes assigned to each patient message, allowing for multiple codes assigned to a single message depending on content. Each team member independently coded the same message narrative line by line in Atlas.ti (ATLAS.ti Scientific Software Development GmbH), a qualitative data management program. Messages were coded individually without accounting for message threads on a single subject.

### Study Oversight

The Institutional Review Board of the University of Maryland, Baltimore approved this study. A data and safety monitoring board was designated to review the study procedures and adverse events. After enrollment was closed, errors in consent were found and all participants, both physicians and patients, were asked to sign consent forms again as recommended by the Institutional Review Board. All patients in the final analysis were reconsented.

### Statistics

The frequency of message themes was computed based on coding to categorize messages using Atlas.ti. Baseline characteristics are expressed as mean and standard deviation for continuous variables comparing users versus nonusers with 2-sample *t* tests or frequencies and proportions for categorical variables comparing users with nonusers with chi-square tests. A mixed methods approach was used to determine the effect of patient engagement on HbA_1c_. Using qualitative analysis data, regression models were developed to determine the predicted change in HbA_1c_ for a patient based on the number and theme of messages sent over the 1-year study period. SAS 9.2 (SAS Institute Inc) was used to perform all statistical analyses. A *P*<.05 was considered statistically significant.

## Results

There were 107 patients in this secondary analysis of MDIS. Among intervention participants, 76.6% (82/107) messaged at any time during the year (users), and 25 participants never messaged during the intervention year (nonusers). Males and females were equally represented. Although not statistically significant, participants who messaged (users) had more education, lower baseline HbA_1c_, and lower body mass indexes (BMIs) than nonusers (Table 1). Users were significantly older (53.5 [SD 7.5] years vs 49.6 [SD 8.9] years, *P*=.03) and more likely to be white (62.2% versus 37.8%, *P*=.02) compared to nonusers.

Table 2 shows the 7 self-care behaviors for healthy living with diabetes as recommended by the AADE plus 2 additional messaging domains. Patients sent messages in an average of 4.3 behavior themes throughout the study. Among all participants, 76.6% (82/107) sent messages in at least 1 behavior theme, and each patient sent an average of 38.4 messages over the 1-year treatment period. Patient engagement was highest for glucose monitoring (75/107, 70.1%), medication (71/107, 66.4%), and reducing risks (71/107, 66.4%) themes and lowest for being active (44/107, 41.1%) and healthy coping (63/107, 58.9%). On average, most messages sent per patient were related to glucose monitoring (9.2, SD 14.0) and healthy eating (6.9, SD 13.2), while patients sent few messages about being active (2.2, SD 5.2) or healthy coping (4.4, SD 8.1).

**Table 1:** Baseline characteristics.

Baseline characteristics		Users (n=82)	Nonusers (n=25)	*P* value
**Glycated hemoglobin A_1c_, n (%)**				.78
	7.5 to 8.9%	42 (51.2)	12 (48.0)	
	≥9.0%	40 (48.8)	13 (52.0)	
Age, years, mean (SD)		53.5 (7.5)	49.6 (8.9)	.03
**Sex**				.28
	Male	43 (52.4)	10 (40.0)	
	Female	39 (47.6)	15 (60.0)	
**Race**				.02
	Nonwhite	31 (37.8)	16 (64.0)	
	White (non-Hispanic)	51 (62.2)	9 (36.0)	
Duration of diabetes, years, mean (SD)		7.8 (5.4)	7.6 (4.9)	
**Education, n (%)**				.36
	High school or less	24 (29.3)	11 (44.0)	
	Some college or associates	34 (41.5)	9 (36.0)	
	Bachelor’s degree or higher	24 (29.3)	5 (20.0)	
**Body mass index (kg/m^2^), mean (SD)**		35.7 (7.2)	36.8 (9.9)	.61
	Normal or underweight (16.5 to 24.9 kg/m^2^), n (%)	2 (2.4)	2 (8.0)	
	Pre-obese (25 to 29.9 kg/m^2^), n (%)	18 (22.0)	5 (20.0)	
	Obese class 1 (30 to 34.9 kg/m^2^), n (%)	20 (24.4)	5 (20.0)	
	Obese class 2 (35 to 39.9 kg/m^2^), n (%)	20 (24.4)	5 (20.0)	
	Obese class 3 (≥40 kg/m^2^), n (%)	22 (26.8)	8 (32.0)	

**Table 2:** Mobile communication messages by patient diabetes behaviors over 1-year treatment period.

Messaging domain		Number	Any messages sent		Messages per patient^a^
			% of total	% of users	Mean (SD)
**Domains**					
	Healthy eating	67	62.6	81.7	6.9 (13.2)
	Being active	44	41.1	53.7	2.2 (5.2)
	Monitoring	75	70.1	91.5	9.2 (14.0)
	Medication	71	66.4	86.6	6.1 (9.3)
	Problem solving	70	65.4	85.4	5.0 (8.2)
	Healthy coping	63	58.9	76.8	4.4 (8.1)
	Reducing risks	71	66.4	86.6	4.5 (6.4)
Any of above behaviors		82	76.6	100.0	38.4 (60.6)
Healthy eating, monitoring, medications		60	56.1	73.2	13.9 (20.7)

^a^Mean messages per patient is calculated for all patients in group, both those that did send messages in this theme and those that did not send messages in this theme.

**Table 3:** Effect of domain messaging on hemoglobin A_1c_.

Message domain		Sent no messages by domain		Sent messages by domain		*P* value	
**Healthy eating, n**		40		67			
	Baseline, mean (SD)		9.8 (2.1)		9.4 (1.9)		
	12-month, mean (SD)		8.2 (1.7)		7.6 (1.3)		
	Change, mean (SD)		–1.6 (2.2)		–1.7 (1.7)		.10
**Being active, n**		63		44			
	Baseline, mean (SD)		9.7 (2.0)		9.4 (2.0)		
	12-month, mean (SD)		7.9 (1.6)		7.7 (1.4)		
	Change, mean (SD)		–1.7 (1.9)		–1.7 (1.8)		.60
**Monitoring, n**		32		75			
	Baseline, mean (SD)		9.8 (2.2)		9.5 (1.9)		
	12-month, mean (SD)		8.3 (1.7)		7.6 (1.4)		
	Change, mean (SD)		–1.4 (2.2)		–1.8 (1.7)		.03
**Medication, n**		36		71			
	Baseline, mean (SD)		9.8 (2.1)		9.5 (1.9)		
	12-month, mean (SD)		8.4 (1.6)		7.6 (1.3)		
	Change, mean (SD)		–1.4 (2.1)		–1.9 (1.7)		.01
**Problem solving, n**		37		70			
	Baseline, mean (SD)		10.1 (2.2)		9.3 (1.9)		
	12-month, mean (SD)		8.3 (1.6)		7.6 (1.4)		
	Change, mean (SD)		–1.7 (2.3)		–1.7 (1.6)		.12
**Healthy coping, n**		44		63			
	Baseline, mean (SD)		9.8 (2.1)		9.4 (1.9)		
	12-month, mean (SD)		8.1 (1.6)		7.6 (1.4)		
	Change, mean (SD)		–1.6 (2.1)		–1.7 (1.7)		.21
**Reducing risks, n**		36		71			
	Baseline, mean (SD)		9.9 (2.2)		9.4 (1.9)		
	12-month, mean (SD)		8.2 (1.7)		7.6 (1.4)		
	Change, mean (SD)		–1.6 (2.2)		–1.7 (1.7)		.12
**Message on any behavior, n**		25		82			
	Baseline, mean (SD)		9.8 (2.3)		9.5 (1.9)		
	12-month, mean (SD)		8.5 (1.8)		7.6 (1.3)		
	Change, mean (SD)		–1.2 (2.2)		–1.8 (1.7)		.02

Participants who sent messages about glucose monitoring (*P*=.03) or medication (*P*=.01) decreased their HbA_1c_ significantly more than those who did not send messages related to those themes (Table 3). Individual theme regression models in Table 4 show that sending any messages lowered HbA_1c_ 0.75 percentage points (95% CI 0.13 to 1.36, *P*=.02) compared to sending no messages. Likewise, sending any messages about glucose monitoring was associated with a decrease in HbA_1c_ of 0.62 percentage points (95% CI 0.05 to 1.19, *P*=.03) and sending any messages about medication was associated with a decrease in HbA_1c_ of 0.72 percentage points (95% CI 0.17 to 1.26, *P*=.01). Based on the top 3 significant themes presented in Table 4, the composite of healthy eating, glucose monitoring, and medication was also tested to determine its combined predictive power. Sending any messages about healthy eating, glucose monitoring, or medication combined significantly decreased HbA_1c_ by 0.54 percentage points (95% CI 0.01 to 1.08, *P*=.02) compared to messages not including these themes (not shown in table).

**Table 4:** Effect of domain messaging (both count and dichotomous) on hemoglobin A_1c_.

Message domain	Message count (continuous)			Any message sent (dichotomous)		
	Estimate^a^	95% CI	*P* value	Estimate^a^	95% CI	*P* value
Healthy eating	–0.005	–0.025 to 0.015	.62	–0.469	–1.020 to 0.083	.10
Being active	–0.017	–0.067 to 0.033	.50	–0.146	–0.692 to 0.399	.60
Monitoring	–0.010	–0.029 to 0.010	.32	–0.624	–1.193 to –0.054	.03
Medication	–0.015	–0.043 to 0.013	.30	–0.717	–1.264 to –0.171	.01
Problem solving	–0.014	–0.047 to 0.019	.40	–0.452	–1.018 to 0.115	.12
Healthy coping	–0.020	–0.053 to 0.014	.25	–0.344	–0.886 to 0.198	.21
Reducing risks	–0.025	–0.070 to 0.019	.26	–0.445	–1.010 to 0.120	.12
Any message	–0.002	–0.007 to 0.002	.35	–0.748	–1.363 to –0.132	.02

^a^Point estimates are per message.

## Discussion

### Principal Findings

Among adults with type 2 diabetes, engagement in the portal messaging system of the MDIS was associated with an absolute decrease in HbA_1c_ of 0.75 percentage points. A 0.5 to 1.0 percentage point change in HbA_1c_ is considered clinically significant to reduce risk of comorbid conditions [37,38]; the US Food and Drug Administration requires a 0.4 percentage point change in HbA_1c_ for drug evaluations [39]. Although any sending of messages was related to a reduction in HbA_1c_, glucose monitoring and medication use themes were also associated with decreases in HbA_1c_. Patients sent the most messages on glucose monitoring, medication use, and reducing complication risks themes. The average number of messages sent per patient was highest for glucose monitoring, medication use, and healthy eating themes.

### Self-Care Behaviors and Hemoglobin A1c

The AADE and the ADA provide patients, researchers, and clinicians with current self-care and lifestyle behavior guidelines for the management of diabetes and the prevention of its complications [35,36]. These guidelines, developed from the findings of the UK Prospective Diabetes Study [40], supply individuals with type 2 diabetes the knowledge needed to better understand their disease. Physicians in this study were given current ADA patient care guidelines but were not explicitly told to use them to care for study patients. Our findings support other studies that have shown the benefits of lifestyle interventions on diabetes outcomes [41-45]. In particular, digital health interventions targeting behavior change have shown lower HbA_1c_ levels, lower random [43] and postprandial [44] plasma glucose levels, and lower body weight [43] as well as improved self-efficacy [45].

Among the behavior themes measured, most messages contained either monitoring, healthy eating, or medication themes. Since messages sent regarding the medication and glucose monitoring themes also significantly decreased HbA_1c_, patients may need more education surrounding medication use and monitoring blood glucose to ensure HbA_1c_ goals can be achieved effectively on their own.

### Patient Engagement

Previous studies that assessed patient engagement in telemedicine and digital health interventions showed that race, age, and health literacy all play significant roles in patient participation [46-48]. Racial minorities, older patients, and patients with low health literacy showed the least engagement in telemedicine and digital health interventions [46,48]. In a 3-month mobile health intervention involving adults with type 2 diabetes, Nelson and colleagues [47] found that those who were younger or were diagnosed with type 2 diabetes closer to the start of the intervention displayed higher engagement activities and had more favorable experiences than older individuals or those with a longer diabetes duration. Our results are consistent with others, showing that nonwhite patients were less likely to send messages to assigned CDEs. Likewise, among participants who did not use the messaging portal, most had a high school education or less, perhaps also indicating a lower health literacy rate. However, unlike previous studies, we observed that users of the messaging portal tended to be older than nonusers. This suggests that older age does not imply disengagement from mobile health technology [49-51]. In fact, in a study evaluating the self-efficacy and use of a mobile health diabetes intervention among older adults, we previously concluded that participants experienced high self-efficacy in making changes to manage their diabetes and demonstrated their ability to use the intervention and communicate with educators [52]. We recommend including older adults and nonwhite individuals in mobile technology development with specific aims to evaluate improving patient engagement.

Other studies concluded that patients’ high engagement in digital health interventions was related to feedback received from physicians or assigned caregivers. From this feedback, patients felt more motivated and were able to attain higher self-efficacy [53,54]. Patients in this study who elected to send messages regarding any self-care behavior reported significant decreases in HbA_1c_. Although the influence of CDE messages on patients’ outcomes was not examined, knowing a diabetes educator was available may have improved patient confidence and encouraged them to participate.

### Mixed-Methods Approach to Analysis

We believe that patient engagement in an intervention cannot be determined simply by a quantitative value but must also include qualitative data that demonstrates the effectiveness of the intervention from the participants’ perspectives. To accurately interpret the extensive data collected from digital health studies, it is important to include a qualitative component [55], as information on individual experience influences the effectiveness of the intervention. We used a mixed-methods approach to evaluate patient engagement data for participants in MDIS. We identified coding themes reflecting patient messages sent to CDEs and analyzed these themes against changes in patient HbA_1c_ values. Results of this study reinforce findings from previous mobile health investigations that use a mixed-methods approach to examine data, collecting self-care behavior and self-efficacy data to measure outcomes [47,56,57]. These studies add valuable knowledge about the usability of digital health applications for the management of diabetes and reveal areas lacking in development that, if revised, could enhance patient user experience and improve diabetes outcomes. This secondary analysis of the MDIS affirms that it is not enough to simply give patients information about diabetes; patients must also be given actionable items that drive behavior change.

### Strengths, Limitations, and Future Directions

The secondary data analysis is, to our knowledge, the first of its kind. Few previous studies have used a mixed-methods approach to evaluate patient engagement. While prior interventions included a patient messaging component [48] or analysis of self-management behaviors [58], none performed a qualitative evaluation of patient messages that was then used to create models predicting the impact on patient clinical outcomes. Furthermore, previous studies show that although participants preferred to use mobile health applications for diabetes management, currently available apps do not offer functions that would allow proper disease monitoring and management [59,60]. Results of this analysis may help pinpoint behavioral features that could improve existing mobile health technologies and satisfy the lack in functionality.

There are a few limitations of this secondary analysis. One is that although the models give a statistically significant prediction of change in HbA_1c_ based on certain message themes, it cannot be definitively stated that this is a direct result of solely the message content. It is important to consider the other aspects of the intervention, such as tracking data, accessing the learning library, or receiving directed care from their primary care physicians as also potentially influencing the patient’s outcome. Also, engagement was not randomized, so there is potential for confounding.

It is also important to note that based on the structure of the program, some patients engaged in external email and phone messages with the CDEs that are not in the portal message records; without knowing the content of these messages, it is impossible to get a complete picture of patient engagement over the year of the study. Furthermore, the role that messages from the diabetes educators play in patient outcome is unknown. While a future analysis may explore the impact of CDEs on patient outcomes, this analysis cannot account for the influence of the content of those messages on patient engagement or overall patient outcomes.

Since each message was analyzed and coded individually, we did not account for message threads. A conversation spanning several messages could have been counted each time the patient mentions the subject when really it is all part of the same conversation on the subject. Our analysis of dichotomies may be based on more tenable assumptions than the analysis per message.

### Conclusion

In this study, messages sent in the combined healthy eating, monitoring, and medication themes or monitoring and medication themes separately significantly improved HbA_1c_ over the study period. Our results provide insight into the importance of health provider feedback and essential self-care behaviors that require greater emphasis when developing mobile health technologies for diabetes populations.

## Abbreviations

AADE: American Association of Diabetes Educators
ACCORD: Action to Control Cardiovascular Risk in Diabetes
ADA: American Diabetes Association
BMI: body mass index
CDE: certified diabetes educator
DSME: diabetes self-management education
HbA1c: glycated hemoglobin A1c
MDIS: Mobile Diabetes Intervention Study
